# Social Disparities in Cardiometabolic Health in Czechia and Venezuela Using the Allostatic Load Model

**DOI:** 10.1093/eurpub/ckac130.095

**Published:** 2022-10-25

**Authors:** A Polcrova, R Nieto-Martinez, JI Mechanick, GA Maranhao Neto, MM Infante-Garcia, H Pikhart, M Bobak, JR Medina-Inojosa, JP Gonzalez-Rivas

**Affiliations:** 1 RECETOX, Faculty of Science, Masaryk University, Brno, Czechia; 2 International Clinical Research Centre, St Anne's University Hospital, Brno, Czechia; 3 FISPEVEN, Caracas, Venezuela; 4 Department of Global Health and Population, Harvard TH Chan School of Public Health, Boston, USA; 5 LifeDoc Health, Memphis, USA; 6 The Marie-Josée and Henry R. Kravis Center, Icahn School of Medicine at Mount Sinai, New York, USA; 7 Department of Epidemiology and Public Health, University College London, London, UK; 8 Division of Preventive Cardiology, Mayo Clinic, Rochester, USA

## Abstract

**Background:**

Subjects with lower socioeconomic status (SES) are exposed to higher levels of environmental stressors. The cumulative effects of chronic stressors on cardiometabolic health can be evaluated using the allostatic load (AL) score. Despite the accepted social gradient, clear relationships between social determinants and cardiometabolic health in populations with different socio-cultural contexts have been rarely explored. This study aimed to compare the relationships of social determinants with AL in different socioeconomic contexts: unstable Venezuela (VE) and stable Czechia (CZ).

**Methods:**

25-64 years old subjects from two cross-sectional population-based samples from CZ (2013-2014, n = 1579, 56% females) and VE (2014-2017, n = 1652, 70% females). The AL score (scaled 0-8) was calculated using 8 cardiometabolic biomarkers (BMI, waist circumference, systolic and diastolic blood pressure, total and HDL-cholesterol, triglycerides). Social characteristics included education in both countries, household income in CZ, and composite measure of SES based on source of income, household head's profession, motheŕs education, and housing conditions in VE. Ordinal regression was performed separately in men and women.

**Results:**

In CZ, men and women with low education and women with low income were more likely to have higher score of AL compared to those with high education and income (OR 1.45, 2.29 and 1.69). In VE, women with low education and low SES were more likely to have higher AL (OR 1.47 and 1.51), while men with low education and low SES were less likely to have higher AL (OR 0.64 and 0.61), compared to those with high education and high SES. Independently of age, sex, and socioeconomic characteristics, Venezuelans were more likely to have higher AL than Czechs.

**Conclusions:**

Associations of social position indices and cardiometabolic health (proxied by AL) differed between CZ and VE, most likely reflecting differences in social environment between the countries.

**Key messages:**

- Social gradients in cardiometabolic health differ among social environments.
- Social gradients in cardiometabolic health differ among sexes.

